# A Case of Thenar Hammer Syndrome

**DOI:** 10.7759/cureus.28047

**Published:** 2022-08-15

**Authors:** Shreyan A Patel, Viraj Munshi, Keenan Bayrakdar, Adam Guyer

**Affiliations:** 1 Internal Medicine, Edward Via College of Osteopathic Medicine, Blacksburg, USA; 2 Internal Medicine, LewisGale Medical Center, Salem, USA; 3 Interventional Radiology, LewisGale Medical Center, Salem, USA

**Keywords:** cyanosis, deep palmar arch, distal radial artery, digital ischemia, thenar hammer syndrome

## Abstract

Thenar hammer syndrome (THS) is characterized by vascular injury and subsequent digital ischemia from acute high-energy trauma or repetitive low-energy trauma to the thenar eminence of the palm. Here, we report the case of a 41-year-old male construction worker who presented with unilateral, cold, painful, and blue-colored fingertips in his left hand. Angiography of his left upper extremity showed abrupt occlusion of the radial artery at the level of the radial styloid process with a poorly developed but patent deep palmar arch, consistent with THS. The ulnar artery and superficial palmar arch were both patent. He had moderate symptomatic relief with administration of low-dose endovascular fibrinolytics, anticoagulation therapy, and a calcium channel blocker during his stay in the hospital and was discharged home on dual antiplatelet therapy.

## Introduction

Thenar hammer syndrome (THS) is a rare condition caused by vascular insufficiency of the distal radial artery and deep palmar arch [1]. The first case of THS was described as traumatic thenar ischemia by Wandtke et al. in 1976 [2]. THS was named due to the mechanism by which this condition arises: using the thenar eminence of the palm to push, pound, squeeze, or twist objects [1]. Occupationally predisposed individuals, such as those working in the roofing, carpentry, construction, mechanic, and farming industries among others, are at higher risk for THS; they frequently use their hands as a substitute for a hammer as well as hand-held vibrating equipment [3].

## Case presentation

A 41-year-old right-handed male construction worker with a past medical history of hypertension and smoking for the past 15 years presented to the emergency department for progressive worsening of cold, painful, and blue-colored fingertips in his left hand for the past three days. He had been having increased cold sensitivity in the fingertips of his left hand intermittently for the past three weeks. He described the pain as a severe throbbing sensation that kept him awake at night. He had several similar episodes in the recent past and was presumptively diagnosed with Raynaud’s phenomenon for which he was most recently started on acetylsalicylic acid, nifedipine, and topical nitroglycerin. His other home medications included losartan, naproxen, and oxycodone/acetaminophen as needed for severe pain. He denied recent or remote injury, fever, chills, chest pain, shortness of breath, abdominal pain, and rashes.

On admission, he was afebrile, hypertensive with a blood pressure of 158/93 mmHg, had a heart rate of 87 beats per minute in sinus rhythm, and had a respiratory rate of 18 breaths per minute with 97% oxygen saturation on room air. On physical examination, all five digits of the left hand were cold to touch, and he had purplish-blue discoloration beyond the distal interphalangeal joints. Capillary refill was less than two seconds in all five digits, and a radial pulse was palpable on the left hand. There was full range of motion in the left arm and hand. There were no abnormalities noted on physical examination of the right arm and hand.

Complete blood count and comprehensive metabolic panel were both unremarkable. Coagulation studies were also normal. His home medications were discontinued, and he was started on intravenous nitroglycerin, labetalol, and verapamil for blood pressure control and peripheral vasodilation. His pain was managed with intravenous fentanyl. He was also anticoagulated with intravenous heparin.

**Figure 1:**
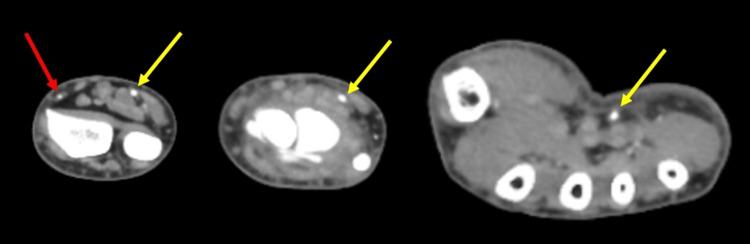
Composite image of CTA of the left upper extremity proximal to the RSP (left), at the level of the RSP (middle), and distal to the RSP (right). The radial artery (red arrow) is patent proximal to the level of the RSP with non-visualization distally. The ulnar artery (yellow arrow) is patent at all levels. CTA: computed tomography angiography; RSP: radial styloid process

Computed tomography angiography (CTA) of the left upper extremity (LUE) (Figure 1) showed abrupt occlusion of the radial artery at the level of the radial styloid process, which was previously patent on a recent CTA of the LUE performed during one of his prior emergency department visits for similar symptoms. The deep palmar arch was poorly developed but patent. The superficial palmar arch was patent. The proximal digital arteries were patent to the level of the proximal interphalangeal joints, but there was poor distal digital perfusion of the first, second, and fifth digital arteries. The left subclavian, axillary, brachial, radial, and ulnar arteries were all patent to the level of the wrist. Arteriography did not show any evidence of occlusion.

A trial of low-dose endovascular fibrinolytics and vasodilators was slowly infused, and the patient reported moderate improvement in symptoms with overnight pharmacotherapy. He was discharged from the hospital the next day with close outpatient follow-up. He was initiated on dual antiplatelet therapy, namely low-dose acetylsalicylic acid indefinitely for life and at least one month of clopidogrel, with continued use if symptoms persisted. He was advised to keep the left hand as warm as possible and to avoid repetitive microtrauma. Above all, he was counseled on the importance of smoking cessation.

## Discussion

THS refers to obstructive injury of the distal radial artery, which forms the deep palmar arch [1]. The severity of symptoms depends on the amount of arterial damage, extent of collateral blood flow between the palmar arches and their branches, and the intrinsic vascular anatomy of the volar surface of the hand [4,5]. THS presents with classic symptoms of pain, numbness, paresthesia, cold sensitivity, and cyanosis [5]. Digital ischemia with progressive ulceration and tissue necrosis may necessitate amputation [1,5].

The pathogenesis of THS involves intimal and medial degeneration with subsequent thrombosis and embolization to the digital arteries [5]. THS may involve aneurysmal changes associated with intimal hyperplasia and fragmentation of the internal elastic lamina [6]. This results in a classic corkscrew appearance of the artery with alternating ectasia and stenosis [6]. Aneurysm formation may also be identified by the characteristic yin yang phenomenon on ultrasound imaging, which refers to bidirectional blood flow within a true aneurysm or pseudoaneurysm [6].

THS is less common than its counterpart, hypothenar hammer syndrome (HHS), which manifests with the same pathogenesis but involves the other side of the palm [1]. While THS predominantly affects the thumb and index finger, HHS typically affects the third, fourth, and fifth fingers due to obstructive injury of the distal ulnar artery and superficial palmar arch [1]. The prevalence of HHS is less than 1% in the general population; the prevalence of THS is lower than that [1,7]. The simultaneous diagnosis of THS and HHS is an even rarer phenomenon [4].

The differential diagnosis for THS also includes thromboangiitis obliterans (TAO), Raynaud’s phenomenon, hand-arm vibration syndrome (HAVS), and cryoglobulinemia. TAO, also known as Buerger’s disease, can present with distal digital ulcerations and cold sensitivity, but symptoms usually begin with claudication in the lower extremities [7]. Raynaud’s phenomenon classically presents with arterial vasospasm followed by a hyperemic phase, which is absent in THS [7]. It can be difficult to distinguish THS from HAVS because both conditions are associated with occupational exposure to hand-held vibrating equipment. HAV initially affects the tips of fingers with the greatest vibratory exposure and progresses to affect the more proximal aspects of fingers over time [8]. In comparison, THS presents more acutely with a severe stage of vascular compromise in the digits receiving blood flow from the distal radial artery and deep palmar arch [1]. Systemic diseases, such as cryoglobulinemia, tend to manifest with bilateral symptoms [7]. 

Our patient had a predisposition for developing THS due to his occupation as a construction worker and 15-year history of smoking. His arteriography results elucidated poor distal perfusion of the first, second, and fifth digital arteries although he was symptomatic in all five fingers of his left hand. His symptoms in the thumb and index finger can be explained by the abrupt occlusion of the radial artery at the level of the radial styloid process, which is consistent with THS. However, there was no arteriographic evidence that definitively demonstrated concurrent HHS. As a result, it is more likely that he was experiencing symptoms in the remaining medial digits due to the intrinsic vasculature of his palmar arches and insufficient collateral blood flow; there is high variability of the hand arteries from one person to another [3-5]. Although he had episodic symptoms in the past that resolved on their own, his symptoms during this episode were constant and progressively worsening without a hyperemic phase, making his presumed diagnosis of Raynaud’s phenomenon unlikely [7]. There may also be a component of HAVS contributing to his clinical presentation, but the acuity of his symptoms more likely suggests THS [8]. HAVS is the result of vascular changes that occur over a longer duration of time and affects and the fingertips more uniformly [8]. The unilaterality of his symptoms excluded systemic diseases, such as cryoglobulinemia [7]. Isolated involvement of the left hand also effectively rules out TAO due to lack of symptoms in the lower extremities [7].

THS typically occurs in the dominant hand owing to physiologic efficiency compared to the non-dominant hand [3]. For example, a right-handed individual typically prefers to use his or her right hand while hammering; however, this may not always be the case [8]. Although our patient was right-handed, he was symptomatic only in his left hand. He used his non-dominant left hand preferentially to perform some of his tasks as a construction worker. Similarly, the vibratory machinery used as part of his occupation may have impacted his left hand more than his right hand. It can be speculated that he may have asymptomatic arteriographic signs of THS, HHS, or HAVS in his dominant right hand as well.

Treatment options for THS include conservative and surgical therapy. First-line conservative therapy entails close monitoring, smoking cessation, use of calcium-channel blockers, use of anticoagulation therapy, and avoiding further injury to the hand [4]. Surgical interventions include thrombolysis, cervical sympathectomy, arterial ligation, and resection of the artery with interposition grafting [4]. Most cases can be treated with conservative therapy; patients requiring surgical treatment typically have some degree of incomplete palmar arch anatomy [4,7]. Although arteriography did not show evidence of occlusion, our patient had moderate symptomatic relief after administration of low-dose endovascular fibrinolytics, anticoagulation therapy, and a calcium channel blocker. As a result, there could have been microemboli that traveled distally to the fingertips and caused the cyanosis. Our patient tolerated conservative medical therapy well.

## Conclusions

THS is a rare clinical condition caused mainly by repetitive microtrauma to the thenar eminence of the palm with subsequent vascular insufficiency of the distal radial artery and deep palmar arch. It is frequently seen in occupationally predisposed individuals. THS is a reversible cause of hand ischemia, so early intervention can significantly reduce morbidity and decrease the risk for amputation. As a result, it is important to take into consideration occupational and recreational history to make the correct diagnosis.
